# Lumbar discal cyst causing bilateral radiculopathy

**DOI:** 10.4103/2152-7806.77026

**Published:** 2011-02-23

**Authors:** Kwak Hyung-Jun, Kim Dae-Yong, Kim Tae-Ho, Park Ho-Sang, Kim Jae-Sung, Jang Jae-Won, Lee Jung-Kil

**Affiliations:** Department of Neurosurgery, Dong Gwang Ju Woori Hospital, Gwangju, Korea; 1 Department of Neurosurgery, Gwang Ju Woori Hospital, Gwangju, Korea; 2 Department of Neurosurgery, Chonnam National University Hospital, Gwangju, Korea

**Keywords:** Bilateral, discal cyst, lumbar spinal stenosis, radiculopathy

## Abstract

**Background:**

Discal cyst is a rare lesion that can result in clinical symptoms typical of disc herniation manifesting as a unilateral single nerve root lesion. To the best of the authors’ knowledge, this is the first reported case of discal cyst resulting in bilateral radiculopathy.

**Case Description:**

A 48-year-old female presented with bilateral sciatica and neurogenic claudication for 3 months. Magnetic resonance imaging revealed an extradural cystic lesion compressing the ventral aspect of the thecal sac at the level of the L3-L4 intervertebral disc. The lesion showed low and high signal intensities on T1- and T2-weighted images, respectively. Total excision of the cyst was achieved after a left hemipartial laminectomy of L3, and an obvious communication with the disc space was found. Bilateral sciatica was immediately resolved after surgery, and was sustained at the two-year follow-up. The histological diagnosis was consistent with a discal cyst.

**Conclusions:**

Although a discal cyst is extremely rare, the possibility of a discal cyst should be considered in differential diagnosis of patients with radiculopathy, particularly when encountering any extradural mass lesion ventral to the thecal sac. Surgical resection is the most employed therapeutic method for symptomatic lumbar discal cysts.

## INTRODUCTION

Low back and sciatic pain is commonly caused by degenerative conditions such as lumbar disc herniation or spinal stenosis. The discal cyst, which has distinct connection to the corresponding intervertebral disc in the spinal canal, is a less common etiology of a lumbar radiculopathy.[2,7,14] We recently encountered a case of discal cyst in which clinical and imaging features differed from those of previous reports. A brief review of previously reported discal cysts in the medical literature is also presented.

## CASE REPORT

A 48-year-old female presented with low back pain radiating to both buttocks and the posterior thigh for 3 months. She also suffered from neurogenic intermittent claudication (NIC) within 10 minutes. The straight leg-raising test was positive at 60 degrees on both sides. However, sensory or motor of the lower extremity was normal.

**Figure 1:**
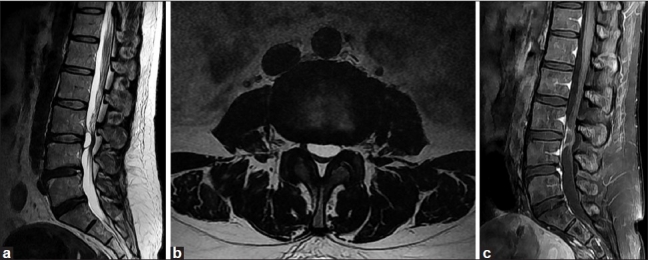
Sagittal (a) and axial (b) T2-weighted MRI demonstrating a cystic lesion at the level of L3-4 intervertebral disc. In sagittal (c) T1-weighted MRI, rim enhancement of the lesion was appreciated after gadolinium administration

**Figure 2:**
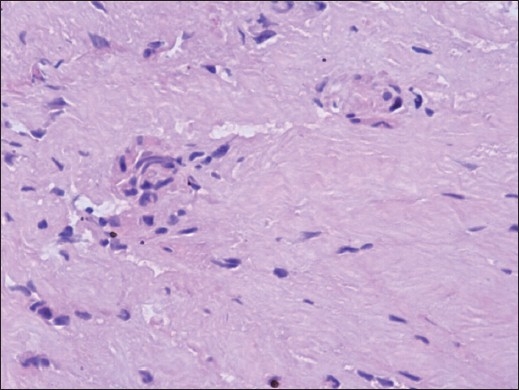
Histopathological examination of the cyst wall revealing fibrous connective tissue without lining cell (H and E, ×200)

**Figure 3:**
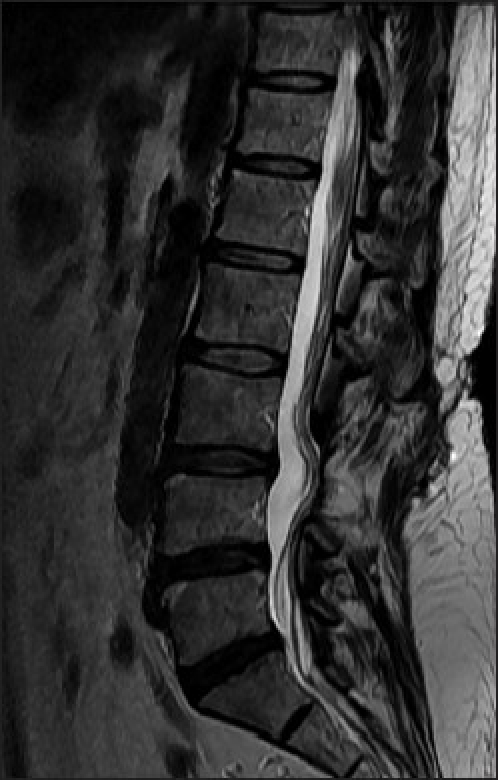
T2-weighted sagittal MRI images at 14-month follow-up after surgery. No recurrent cyst and no evidence of progression of degenerative change in the L3-4 disc were observed

Magnetic resonance imaging (MRI) demonstrated a cystic lesion measuring 6 × 16 × 16 mm with low-signal intensity on T1-weighted imaging and high-signal intensity on T2-weighted imaging at the level of the L3-L4 disc. The cystic mass, located in the ventral aspect of the extradural space, displaced the thecal sac dorsally [Figure 1a and b]. Rim enhancement of the lesion was appreciated after administration of gadolinium [Figure 1c]. The L3-L4 intervertebral disc appeared to have mild degeneration. After L3-L4 laminotomy, a tense dark blue-colored cystic lesion compressing the entire thecal sac was encountered. The cyst was found to be mildly adherent to both the thecal sac and the posterior longitudinal ligament (PLL). The bloody serous fluid was aspirated from the cyst. The cyst was traced back to its stalk, which communicated with the L3-L4 disc by means of a central annular tear. The cyst and stalk were excised completely at the base of the connection. Coagulation of the PLL surrounding a round defect was performed; however, the disc space was not entered. The patient achieved complete pain relief and was allowed to walk on the day after surgery. Histopathological examination of the cyst revealed thick fibrous connective tissue interspersed with areas of chronic inflammation. However, there was no evidence of specific lining cell layers or disc material [Figure 2]. MRI obtained 14 months after surgery showed no evidence of recurrence or progression of disc degeneration [Figure 3]. The patient has remained asymptomatic during a two-year follow-up period.

## DISCUSSION

Discal cyst is extremely rare. To the best of our knowledge, only 57 cases of discal cyst including our case, have been reported in the literature.[1–9,11–13,15–17] Fifty-one patients (89.5%) were males and 6 (10.5%) were females, and ranging age from 13 to 73 years (mean 33.8, median 31.1). The most common cyst locations were L4-L5 in 21 patients (36.8%), followed by L5-S1 in 16 patients (28.1%), L3-L4 in 12 patients (21.1%), L2-L3 in 5 patients (8.8%) and L1-L2 in 3 patients (5.2%). Clinical symptoms of discal cyst are indistinguishable from those of a typical disc herniation manifesting as a unilateral lumbar radiculopathy.[2,12] Patients may suffer from NIC if the cyst becomes large enough to significantly compromise the diameter of the spinal canal. The present case is the only patient who presented with bilateral radiating pain and NIC.

Pathogenesis of discal cysts remains unknown. Two hypotheses for the development of discal cysts have been proposed. Chiba *et al*,[2] hypothesized that an epidural hematoma is initially formed from hemorrhage of the epidural venous plexus that occurs in the space between the peridural membrane and the vertebral body, and discal cysts form most likely as a consequence of impairment of hematoma resorption. However, this theory does not explain the communicating stalk between the intervertebral disc and the cyst. Kono *et al*,[12] proposed focal degeneration of an intervertebral disc with fluid production, similar to formation of meniscal cysts in the knee. Histologic findings from the cyst wall in a previous series and in our case demonstrated fibrous connective tissue without synovial lining cells, which supports this hypothesis.

MRI is the modality of choice for diagnosis of discal cyst. The cyst is round or oval in shape, with a low-intensity signal in T1-weighted images and a high-intensity signal in T2-weighted sequences that is consistent with a cyst containing liquid. This signal can vary depending on the proteinaceous concentration of fluid, or even the presence of blood. The peripheral rim of the cyst is enhanced on contrast-enhanced MRI.[2,11,13] The cyst is a ventrolateral extradural lesion attached to a lumbar disc, and occasional extension into the lateral recess. In most reported cases, MRI has also revealed minimal degeneration of the involved disc. A connecting channel between the cyst and corresponding intervertebral discs can be demonstrated by discography and CT discography. On discography, contrast medium rapidly flowed into the cyst through a thin channel from the disc cavity, and severe radiating pain was simultaneously reproduced in the affected leg.[11] Although discography can definitively diagnose the discal cyst, we did not perform it due to MRI findings showing a high index of suspicion of discal cyst. Moreover, intraoperative findings of the apparent connection between the corresponding disc and the cyst also make it possible to differentiate discal cysts from other cysts.[6,11,15]

Therapeutic guidelines have not been established. Spontaneous regression of the cyst has been reported,[3,5] and intracystic steroid injections with successful resolution have also been attempted.[11] However, surgical excision of the cyst has been employed in the majority of symptomatic discal cysts, and is highly effective for pain relief.[2,6,13,15] An additional discectomy along with the associated cyst might depend on the rate of corresponding disc degeneration. Several authors emphasized the potential benefits to computed tomography or fluoroscopic-guided percutaneous aspiration of discal cyst.[4,8,11] Percutaneous aspiration could be another initial option for discal cysts due to potential advantages including faster recovery, low complication rates, and avoidance of general anesthesia. In patients who failed initial percutaneous trial or recurred cases, surgical excision could be performed subsequently. Because of limited results of percutaneous aspiration by a small sample size and short-term follow-up period, careful analysis and follow up with additional cases are required for establishment of a proper therapeutic strategy.

Kono *et al*,[12] have suggested that the discal cyst could not develop in the midline because the PLL prevents the cyst from developing dorsally to it. In almost all reported cases, cysts were located between the midline of the posterior vertebral bodies and the pedicles. However, in three cases, including ours, cysts that crossed the midline were reported.[7,10] A median septum, connects ventrally with the thickened periosteum and dorsally with the PLL. However, it is not present in the intervertebral disc space in which the PLL is strongly attached to the annulus fibrosus. Considering the discal cyst crossing the midline, we suggest two possible pathways for growth of discal cysts: 1) when discal cysts arise from the area covered by peridural membrane only, they may grow between the midline of the posterior vertebral bodies and the pedicles, and spread to the lateral recess, causing unilateral root symptoms and 2) when discal cysts originate from the area covered by the PLL, perforation of annulus fibrosus, peridural membrane, deep PLL, and superficial PLL are required for bilateral growth of a midline crossing discal cyst. Under these circumstances, bilateral radiculopathy and NIC are likely to occur.

In summary, we present a rare case of a lumbar discal cyst with bilateral radiculopathy. Our case suggests that a discal cyst may cross the midline, resulting in bilateral radiculopathy and NIC. Surgical excision of the cyst offered immediate symptomatic improvement, which was sustained at the two-year follow-up.
