# Supplementary material for: Lumbar discal cyst causing bilateral radiculopathy
Source: Surg Neurol Int. 2011 Feb 23;2:21. doi: 10.4103/2152-7806.77026 (PMC3050063; doi:10.4103/2152-7806.77026)
Supplement: Related texts

# Article commentary: Lumbar discal cyst causing bilateral radiculopathy

**Authors:** Ron Pawl

**Affiliations:** University of Illinois, Chicago (Ret.), Center for Pain Treatment and Rehabilitation, Lake Forest Hospital, Lake Forest, Illinois USA. Email: ron@pawl.com

Discal cyst is certainly a rare pathology. In addition to the two origins of discal cysts noted by the authors,[4] two additional possible origins have been proposed. Kobayashi *et al*, have proposed that one origin is the incomplete absorption process of a herniated disc,[3] while Gadan *et al*, have proposed a traumatic process to the posterior longitudinal ligament, noting that their ten cases were found in young athletic subjects and that the microscopic findings were compatible with such a diagnosis.[1] Differentiating between the origin of a discal cyst seems important in determining the best surgical approach and when to leave the disc space alone in the process, as was done by the authors in the current case presentation.

Also, from the standpoint of surgical technique, Kim and Lee,[2] in their paper regarding 14 cases, have noted the value of CO2 laser-assisted ablation of the discal cyst.

## References

[18] Gadan BR, Le Gars D, Sevestre H, Debussche C, Galibert P, Rosat P (1992). Non-discal sciatica secondary to a cystic lesion of the posterior longitudinal ligament: 10 cases. Presse Med.

[19] Kim JS, Lee SH (2009). Carbon dioxide (CO2) laser-assisted ablation of lumbar discal cyst. Photomed Laser Surg.

[20] Kobayashi S, Takeno K, Uchida K, Yayama T, Nakajima H, Miyazaki T (2010). Pathogenesis of the discal cysts communicating with an adjacent herniated disc. Histological and ultrastructual studies of two cases. Joint Bone Spine.

[21] Kwak HJ, Kim DY, Kim TH, Park HS, Kim JS, Jang JW Lumbar discal cyst causing bilateral radiculopathy. Surg Neurol Int 2011 in press.

# Article commentary: Lumbar discal cyst causing bilateral radiculopathy

**Authors:** Nancy E. Epstein

**Affiliations:** Department of Neurological Surgery, The Albert Einstein College of Medicine, Bronx, NY 10467 and Neurosurgical Spine and Education, Winthrop University Hospital, Mineola, NY 11501, USA. Email: dch3@columbia.edu

## A review of case report: Lumbar discal cyst causing bilateral radiculopathy

The authors present a case report concerning a 48-year-old female with bilateral sciatica and neurogenic claudication of 3 months duration secondary to an MR-documented lumbar discal cyst at the L3–L4 level. Low signal T1 and high signal T2 MR studies documented the anatomy of the cyst which was subsequently adequately excised, without the disc space being entered, through a left hemipartial L3 laminectomy. Postoperatively, the patient’s symptoms totally resolved and she remained asymptomatic after two postoperative years.

When the authors reviewed the literature, they found references which included 57 discal cysts. Most patients were males, averaging 34 years of age, and had discal cysts predominantly at the L4–L5 and L5–S1 levels (64.9%). Symptoms typically included unilateral radiculopathy, unlike the present case which involved bilateral radiculopathy and neurogenic claudication. The authors quoted different hypotheses for the pathogenesis of discal cysts. Although the first hypothesis invoked epidural hematoma as the cause of the cyst, it did not explain why/how the “communicating stalk” developed between the disc and the cyst. The second, more likely hypothesis, offered focal degeneration of the disc with resultant “fluid production”; anatomical data demonstrating a fibrous cyst without a synovial lining supported this hypothesis.

However, the literature offers additional hypotheses regarding the pathoanatomical evolution of discal cysts, while also providing multiple differential diagnoses. In a study by Kobayashi *et al*., discal cysts communicated with an adjacent disc herniation on both MR and CT studies; this finding was confirmed intraoperatively.[7] The lesion located within the epidural compartment, compressed a single nerve root. Following excision of the cyst, histology and electron microscopy revealed residual disc tissue within the cyst wall. Their hypothesis was that macrophages contributed to the formation of a cyst following resorption of herniated disc tissue; subsequent hemorrhage within the cyst wall was the source of the serous fluid that replaced the resorbing disc. In another study by Cho *et al*., lumbar intraspinal epidural ganglion cysts contributed to low back/leg pain.[2] In another study by the same authors, two male patients with unilateral radiculopathy attributed to “rounded” intraspinal cysts on MR (hypointense on T1 and hyperintense on T2) not associated with significant disc herniations, also had ganglion cysts.[3] Both patients exhibited significant degeneration of the ligamentum flavum. Furthermore, the authors also acknowledged that both ganglion and synovial cysts can present as juxtafacet cysts, and that differentiating between them may be difficult.

Gas-filled intradural cysts may also contribute to unilateral radiculopathy.[8] In one case study, a 67-year-old female with marked right thigh pain had an enhanced MR scan which documented a cystic, gas-containing lesion within the spinal canal at the L2–L3 level. The CT-discogram documented contrast filling the cerebrospinal fluid pathways around but not penetrating the cyst. Removal of the cyst was accomplished by dissecting away the firmly adherent nerve root (from the capsule), defining the margin between the cyst and dura, and ultimately by removing the cyst and its discal contents.

In yet another study, an intradural disc herniation containing gas, which appeared as a ring-enhancing “cystic” intradural lesion at the L3–L4 level on a contrast-enhanced MR study, was surgically excised.[1] A gas-filled intradural cyst contributing to lumbar radiculopathy was also seen in a further study that discussed the similarities of gas-containing pseudocysts, herniated intradiscal gas, and free gas within the epidural compartment.[6]

What diagnostic studies best document discal cysts? Although CT-discography readily documents the connection between the disc space and the cyst *via* an attenuated channel, it poses multiple inherent risks which include durotomy, root injury, infection, allergic reactions, etc. Notably, several non-invasive modalities, including MR and CT-based studies, with/without intravenous contrast, would likely adequately confirm the location and etiology of many of these lesions.

Although there are multiple therapeutic options offered to manage discal cysts, open surgical procedures, and not the minimally invasive ones, offer the most versatile therapeutic alternatives. Certainly, non-surgical treatment in minimally and/or asymptomatic patients may be a reasonable alternative, particularly since some discal cysts will spontaneously resorb.

However, other minimally invasive approaches such as intracystic steroid injections, or fluoroscopic/CT-guided cyst aspiration, are likely to fail. Reasons for failure include first, their inability to address the more complex pathology (e.g. disc herniations, synovial cysts, etc.). Second, the thecal sac and/or nerve roots, located dorsal to the discal cyst (with/without disc herniations), may be perforated with these percutaneous techniques, resulting in cerebrospinal fluid fistula formation, and/or root trauma. Third, these techniques provide minimal access to potentially more complex pathology, and therefore increase the risk to the patient of incomplete, inadequate cyst decompression, and/or cyst recurrence. Although Dasenbrock *et al*. observed that an L5-S1 discal cyst was successfully aspirated under CT-guidance, they acknowledged that few surgeons were opting for this approach as another study cited cyst recurrence following a similar approach.[4] Although three of four patients with epidural/intradiscal gas were successfully treated with percutaneous needle aspiration of the gas, this could have resulted in inadvertent durotomy and/or other unanticipated local neural trauma.[5] The optimal operation for discal cysts should be individually tailored to the patient’s specific needs. Furthermore, as open procedures offer more flexibility, visibility, and effectiveness, they should not be replaced by minimally invasive alternatives simply because of the smaller size of the incision. It is critical here, that “marketing” of the minimally invasive techniques does not take the place of good judgment and safer surgery.

## References

[22] Chiu LJ, Tsai PC, Chou PC, Kuo TM, Cheng C, Li MJ (2008). A gas filled intradural cyst with intradural disc herniation: A case report. Acta Neurol Taiwan.

[23] Cho SM, Rhee WT, Choi SJ, Eom DW (2009). Lumbar intraspinal extradural ganglion cysts. J Korean Neurosurg Soc.

[24] Cho SM, Rhee WT, Lee SY, Lee SB (2010). Ganglion cyst of the posterior longitudinal ligament causing lumbar radiculopathy. J Korean Neurosurg Soc.

[25] Dasenbrock HH, Kathuria S, Witham TF, Gokasian ZL, Bydon A (2010). Successful treatment of a symptomatic L5-S1 discal cyst by percutaneous CT-guided aspiration. Surg Neurol Int.

[26] Kakitsubata Y, Theodorou SJ, Theodorou DJ, Yuko M, Ito Y, Yuki Y (2009). Symptomatic epidural gas cyst associated with discal vacuum phenomenon. Spine.

[27] Kawaguchi S, Yamashita T, Ida K, Ikeda T, Ohwada O (2001). Gas-filled intradural cyst of the lumbar spine. Case report. J Neurosurg.

[28] Kobayashi S, Takeno K, Uchida K, Yayama T, Nakajima H, Miyazaki T (2010). Pathogenesis of the discal cysts communicating with an adjacent herniated disc. Histological and ultrastructural studies of two cases. Joint Bone Spine.

[29] Sei A, Mizutamari M, Fujimoto T, Taniwaki T, Mizuta H (2009). Gas-filled intradural cysts of the lumbar spine and the possible pathogenesis. Spine J.

# Article commentary: Discal cysts: Rare causes of nerve root compression

**Authors:** Ali Bydon

**Affiliations:** Department of Neurosurgery, Johns Hopkins University, 600 N. Wolfe Street, Meyer 7-109, Baltimore, MD 21287, USA. Email: abydon1@jhmi.edu

A 48-year-old Asian female presented with a 3-month history of bilateral lumbar radiculopathy. An LS spine magnetic resonance imaging (MRI) revealed an L3-4 discal cyst (hyperintense on T2-weighted sequences and hypointense on T1-weighted images with a slight rim enhancement) crossing the midline and causing bilateral nerve root compression. The patient underwent hemilaminectomy for cyst resection, and aspiration of bloody serous fluid. The cyst was traced back to its discal fistulous attachment where an annular tear was discovered and coagulated. The disk space itself was not violated. Clinical and radiographic follow-up, at 12 and 14 months, respectively, revealed excellent symptomatic and cyst resolution.

Degenerative conditions of the lumbar spine are common causes of radiculopathy. A number of rare conditions exist that may present identically; discal cysts, intraspinal extradural cysts that communicate with the intervertebral disc, are a rare cause of radiculopathy. First reported in English in 1999,[7] only less than 100 patients with discal cysts have been published until now, primarily as case reports and small case series.[1] This is the first reported case of a discal cyst causing bilateral symptoms.

Discal cysts are seen most commonly in young Asian men.[1] The fact that discal cysts occur at more rostral levels than disc herniations (the L3-4 level in this case), can present in the absence of additional spinal pathology and most commonly affect young males, may suggest a pathophysiology distinct from that of degenerative conditions.

The etiology of discal cysts is debatable. Some suggestion underlying traumatic epidural hematoma with deficits in its resorption leading to cyst formation.[2] Others believe that focal degeneration of the intervertebral disc leads to leakage of fluid into the spinal canal prompting a marked inflammatory reaction, causing the formation of a pseudomembrane around the fluid.[6] Finally, Marshman *et al*. have argued that discal cysts are synonymous with posterior longitudinal ligament/annulus fibrosus ganglion cysts, likely degenerative in origin.[8]

MRI is the diagnostic study of choice, typically revealing an extradural intraspinal ventrally located cyst, hypointense on T1-weighted and hyperintense on T2-weighted images, with rim enhancement after gadolinium administration (as seen in this case). However, hemorrhagic cysts may be hyperintense on both T1- and T2-weighted images, and the rim of contrast enhancement is not always present. Discography, albeit invasive, is used diagnostically to confirm a connection between the intervertebral disc and the cyst.

The natural history of this condition has yet to be defined, providing little guidance on prognosis. Spontaneous regression of a discal cyst has been reported,[4] as well as regression after intradiscal and epidural steroid injections.[5] Although most reported cases have failed a trial conservative therapy, it is not clear if this represents a publication bias or if discal cysts are less responsive to conservative therapy. Nevertheless, an initial trial of conservative therapy should be pursued in neurologically intact patients.

Treatment options exist, though surgical resection is the most commonly employed modality. Successful CT-guided percutaneous aspiration of discal cysts has been reported.[3] Its proponents point to its many potential advantages: decreased infection rates, avoidance of general anesthesia, and faster recovery. They argue that surgical excision should be reserved for patients who fail percutaneous drainage attempts. However, surgical excision remains the most commonly performed therapeutic modality used for symptomatic discal cysts, with complete irradication of the fistulous connection between the disk and the cyst in order to avoid recurrence.

## References

[30] Aydin A, Abuzayed B, Yildirim H, Bozkus H, Vural M (2010). Discal cysts of the lumbar spine: Report of five cases and review of the literature. Eur Spine J.

[31] Chiba K, Toyama Y, Matsumoto M, Maruiwa H, Watanabe M, Nishizawa T (2001). Intraspinal cyst communicating with the intervertebral disc in the lumbar spine. Spine.

[32] Dasenbrock HH, Kathuria S, Witham TF, Gokaslan ZL, Bydon A (2010). Successful treatment of a symptomatic L5/S1 discal cyst by percutaneous CT-guided aspiration. Surg Neurol Int.

[33] Demaerel P, Eerens I, Goffin J, Wilms G (2001). Spontaneous regression of an intraspinal disc cyst. Eur Radiol.

[34] Dumay-Levesque T, Souteyrand AC, Michel JL (2009). Steroid injection performed with fluoroscopy for treatment of a discal cyst. J Rheumatol.

[35] Jeong GK, Bendo JA (2003). Lumbar intervertebral disc cyst as a cause of radiculopathy. Spine J.

[36] Kono K, Nakamura H, Inoue Y, Okamura T, Shakudo M, Yamada R (1999). Intraspinal extradural cysts communicating with adjacent herniated disks: Imaging characteristics and possible pathogenesis. AJNR Am J Neuroradiol.

[37] Marshman LA, Benjamin JC, David KM, King A, Chawda SJ (2005). “Disca cysts” and “posterior longitudinal ligament ganglion cysts:” Synonymous entities?. Neurosurgery.
